# Mass/Heat Transfer Analogy Method in the Research on Convective Fluid Flow through a System of Long Square Mini-Channels

**DOI:** 10.3390/ma15134617

**Published:** 2022-06-30

**Authors:** Joanna Wilk, Sebastian Grosicki, Robert Smusz

**Affiliations:** Thermodynamics Department, Rzeszow University of Technology, Al. Powstańców Warszawy 12, 35-959 Rzeszow, Poland; sebogr@prz.edu.pl (S.G.); robsmusz@prz.edu.pl (R.S.)

**Keywords:** minichannel, heat transfer, mass transfer coefficient, limiting current method

## Abstract

The paper presents the results of experimental investigations of mass transfer processes with the use of the limiting current technique. This experimental work analyzed the not fully developed entrance laminar region. The tested case refers to the convective fluid flow through a system of nine long, square mini-channels that are 2 mm wide and 100 mm long. The method used in the measurements allows one to determine mass transfer coefficients during the electrolyte flow by utilizing electrochemical processes. The received mass transfer coefficients were applied to the analogous heat transfer case. The Chilton–Colburn analogy between mass and heat transfer was applied. The obtained results, in the form of the dependence of Nusselt number within the function of Reynolds and Prandtl numbers, can be a useful formula in the design and analysis of heat transfer processes in mini heat exchangers.

## 1. Introduction

Heat transfer in mini-channels is an important topic in current science and technology studies. Investigations of heat transfer processes occurring in channels of small, cross section dimensions, i.e., mini-channels, comprise a significant portion of the research conducted within a wide range of applications. Practical mini-channel applications include heat sinks, compact heat exchangers, electronic- and bio- chips, and air conditioning and refrigeration systems, among others. In all applications, the material properties which the heat transfer systems are composed of play a crucial role. The description of heat transfer processes in mini-channels requires a knowledge of the thermophysical properties of both the materials of energy mini-devices and of heat transfer fluids. These properties are primarily thermal conductivity, specific heat, and thermal diffusivity. In most cases, the commercial apparatus allows one to determine the thermophysical properties for various kinds of materials. The exemplary works are [1,2,3,4], where the authors applied classical methods to investigate metals, nanofluids, or composites. However, occasionally unconventional research procedures will be necessary, the following of which can be cited here: unconventional laser flash technique [5], nondestructive real time technique [6], or integral transform method [7] for thermophysical properties determination.

To explore heat transfer processes in mini-channels, certain factors should be considered. The condition of the inner surface of the mini-channel is important. Factors influencing the surface condition are roughness [8] and fouling [9]. Another issue is the type of heat transfer fluid: gas or liquid, single-phase or two-phase fluid flow [10,11,12,13,14]. Single phase heat transfer processes in micro- and mini-channels with the use of various material properties of working fluids were studied in [10,13,14]. In turn, the works [11,12] present the results of the research of new refrigerants condensation in mini-channels.

In addition to thermophysical properties, the heat transfer coefficient *h* plays a significant role in the thermal design of materials of different kinds of mini systems. Determination of *h* on the basis of the analytical solution of the governing equations system is possible only in select cases. Thus, there is a need for other approaches. One is numerical simulation. Recently, many researchers have studied the problem of heat transfer in mini-channels numerically. The exemplary works on numerical investigations of heat sinks with circular and rectangular mini-channels are presented in [15,16]. In turn, article [17] includes a comprehensive review of the mathematical and numerical models used in the thermal analysis of mini-channels.

The second group of methods for determining the heat transfer coefficient in mini-channels are experiments. Most experimental investigations of heat transfer processes are performed with the use of the thermal balance method. The exemplary works include the results of thermal experiments on heat transfer in mini-systems, namely the above-mentioned papers [15,16,17]. An alternative method for determining the heat transfer coefficient during convective fluid flow through mini-channels is the mass/heat transfer analogy method. This method is based on mass transfer measurements and on the analogy between mass and heat transfer processes. The analogy method is useful when the thermal experiment can be difficult to conduct, as is the case with mini-channels, where thermal measurements may be complicated due to small channel dimensions. The results of investigations on mass transfer in mini-systems can be found in [18,19,20,21,22,23,24]. The works [18,19] describe the mass/heat transfer analogy method in application for mini-channels and comprehensive review of the investigations of mass transfer in mini-systems. In turn, the papers [20,21] present the results of mass transfer coefficients measurements in the case of long circular and rectangular microchannels, while the articles [22,23] include investigations of mass transfer in short circular mini-channels. In [24], the authors experimentally studied mass transfer processes in the single long square mini-channel. 

The main goal of the present work was to investigate mass transfer process during the electroactive fluid flow through the system of long square mini-channels. In the present study, the results of measurements of mass transfer coefficients in the system tested are presented.

The obtained results make it possible to determine heat transfer coefficients characterizing analogous thermal cases. As the mass/heat transfer coefficients depend on may factors, including the kind of material of transfer surface (electrode) and its condition, material properties of the fluid transferring the mass/heat, or flow conditions resulting from the geometry of the system (channel square cross section and mini dimensions), these issues have been considered in the presented research. The possibility of applying the analogous thermal results in a wide range of materials in energy technology issues seems to be an important effect of the conducted research.

## 2. Materials and Methods

The method used in the present study to determine mass/heat transfer coefficients by the mass/heat transfer analogy is the electrochemical technique known as the limiting current method. This method involves observation of the controlled ion diffusion at the cathode. Once an external voltage is applied to the electrodes immersed in the electrolyte, the anionic reduction occurs at the cathode and the oxidation process occurs at the anode. The electric current arises in the external circuit as a result of the processes. According to Faraday’s law, the current generated *I* is given by:

(1) I=nFAN,

where *A*—cathode surface area, *N*—molar flux density, *n*—number of transferred electrons in the electrochemical reaction (*n* = 1 for this study), and *F*—Faraday constant, *F* = 96,493 × 10^3^ As/kmol.

Basing on Fick’s and Nernst’s laws, one may write:

(2) $$I=nFAh_{D}\left(C_{b}-C_{w}\right),\;$$

where *h_D_*—mass transfer coefficient, *C_b_*—bulk ion concentration, and *C_w_*—ion concentration at the cathode surface.

In the limiting current method, an increasing current is caused to flow across the electrodes by increasing the applied voltage until a characteristic point is reached. If the anode surface is much larger than the cathode, a further increase in the applied voltage will not lead to increased current intensity; if the limiting current *I_p_* is achieved, the controlled diffusion occurs. Under these conditions, the ion concentration at the cathode *C_w_* approaches zero. Based on the measurement of the limiting current *I_p_*, the mass transfer coefficient can be calculated from the equation:

(3) $$h_{D}=I_{p}/nFAC_{b}.$$

In the present study, a classical system for mass transport measurements using the electrolytic technique was applied. It is a reduction of ferricyanide ions at the cathode and oxidation of ferrocyanide ions at the anode. A solution of sodium hydroxide was used as the background electrolyte. The oxidation-reduction process under convective-diffusion controlled conditions is written as:

(4) $${\left[Fe{\left(CN\right)}_{6}\right]}^{-4}\overset{ox\;\;\;\;\;\;red}{↔}{\left[Fe{\left(CN\right)}_{6}\right]}^{-3}+e.$$

Cathodes and anodes of nickel-have been applied in the experiment.

**Figure 1:**
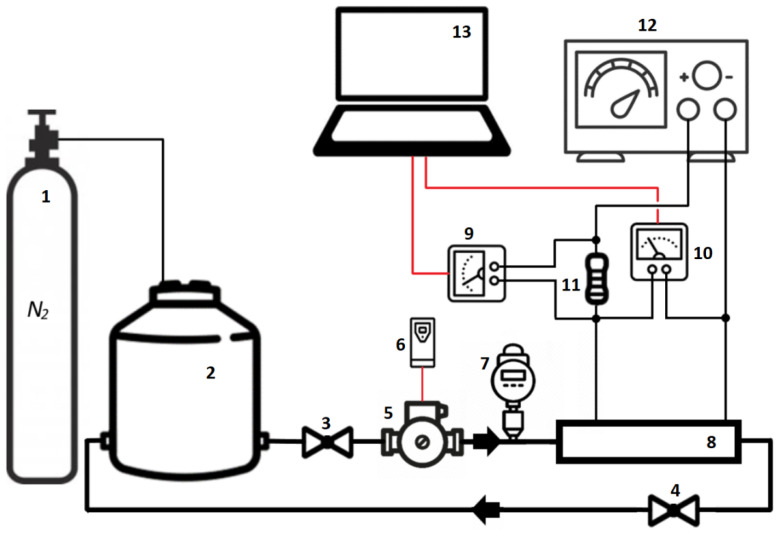
Schematic diagram of the experimental equipment: 1—portable nitrogen gas cylinder; 2—electrolyte reservoir; 3,4—valves; 5—pump; 6—variable-frequency drive; 7—turbine flowmeter, 8—test section with the system of square minichannels; 9,10—laboratory digital multimeters; 11—high precision standard resistor; 12—DC laboratory power supply; 13—computer with data logger.

**Figure 2:**
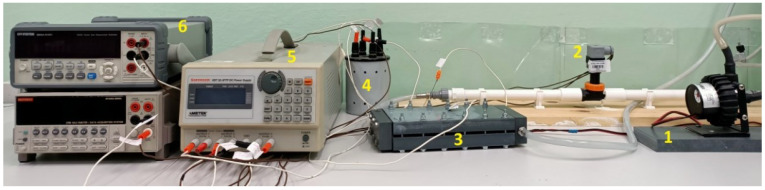
View of the main elements of the test rig: 1—pump, 2—turbine flowmeter, 3—test section with the system of square minichannels, 4—high precision standard resistor, 5—DC laboratory power supply, 6—laboratory digital multimeters.

The mass transfer experiment was performed on a specific stand, the schematic diagram of which is shown in Figure 1. In turn, Figure 2 presents the photo of the main elements of the experimental test rig. Elements of the measurement system which were in contact with the electrolyte were made of materials characterized by chemical resistance. To achieve the controlled diffusion process, the removal of oxygen from the electrolyte is necessary. This was fulfilled by bubbling the electrolyte with nitrogen.

**Figure 3:**
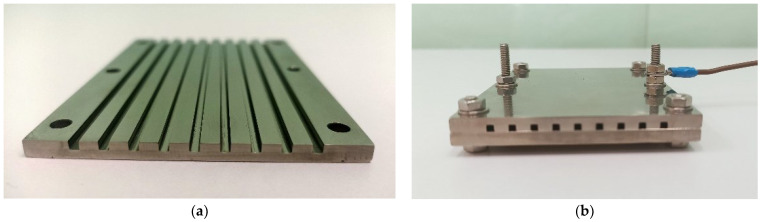
Test section with the investigated mini-channels: (**a**) Cathode of the limiting current method—the inner nickel surface of the square mini-channels; (**b**) The general view of the minichannel’s system.

**Figure 4:**
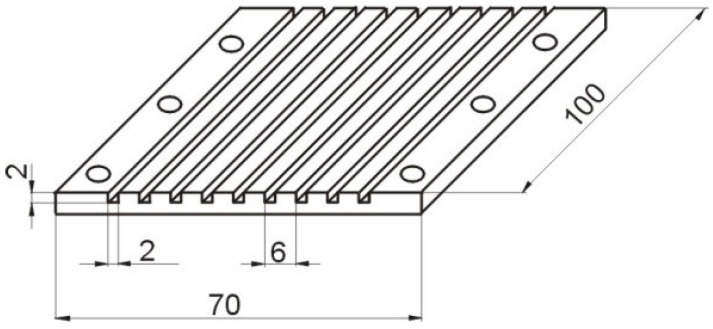
Geometry of the test section with long square mini-channels (dimensions in mm).

The investigated test section consisted of nine square mini-channels having a width of 2 mm (equal to the hydraulic diameter *d_H_*) and length *L* of 100 mm. The nickel inner surface of mini-channels functioned as a cathode. The view and the geometry of the test section with mini-channels are presented in Figure 3 and Figure 4.

The anode with a reaction surface larger than the cathode reaction surface was located behind the cathode in the electrolyte flow direction. Before the electrochemical experiment, the cathode and anode surfaces were specially prepared and polished using the special greasy abrasive compound with different granularities. Such preparation of the nickel surface is performed to enable a smooth surface.

For determining the mass transfer coefficient from Equation (3), measurement of the bulk ion concentration *C_b_* is necessary. As the long channel with a small hydraulic diameter was examined, there could be a change in ion concentration along its length [19]. On the basis of the balance of the electrolyte molar flux and including Equation (3), the ion concentration at the channel outlet *C_out_* is derived from the formula:

(5) $$C_{out}=C_{in}-I_{p}/\left(nF\dot{V}\right),\;$$

where *C_in_*—ion concentration at the mini-channel inlet and $\dot{V}$—volumetric flow rate of the electrolyte. The ferricyanide ion concentration at the mini-channel inlet was measured by the use of iodometric titration. Because the ion concentration is changing over time, the iodometric titration was carried out before each measurement.

Considering Equation (5), the average bulk ion concentration was calculated using the formula:

(6) $$C_{b}=\frac{C_{in}-C_{out}}{ln\left(C_{in}/C_{out}\right)}$$

Parameters of the current were controlled and measured with the use a Sorensen XBT 32-3FTP direct current supply, voltmeters, and standard high precision 1 Ω resistor. The Swiftech MCP655 magnetic drive vane pump forced the flow of electrolyte. To determine the electrolyte velocity, the flow rate was measured by a Kobold turbine flowmeter with a current signal. Investigations were performed for six electrolyte velocities. Two measurements were made for each case. An air conditioning system was utilized to maintain the measurement temperature at 22 °C and thus the thermophysical properties of the electrolyte, kinematic viscosity and diffusion coefficient, were constant.

## 3. Results and Discussion

**Figure 5:**
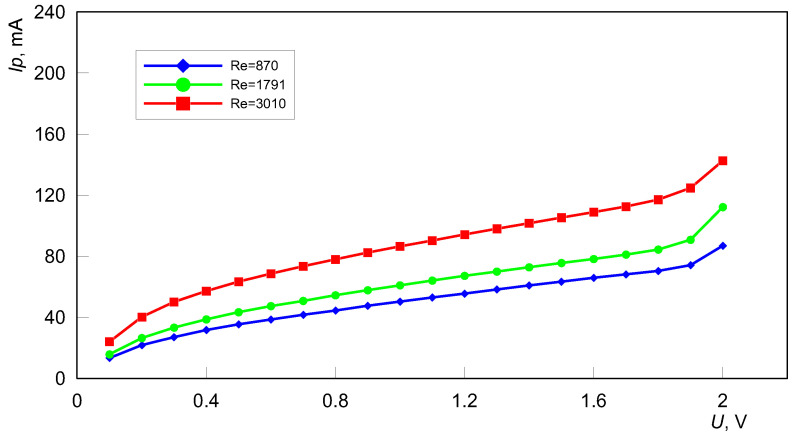
Voltammograms for the reduction of ferricyanide ions at the cathode—the inner surface of the mini-channels.

The base result of the applied electrochemical technique, the limiting current method, is the value of the limiting current reached when the controlled diffusion at the cathode occurs. It is visible on the measurement’s graphs of the current against the applied voltage. In the current-potential behavior, the flat part, i.e., the plateau, represents the limiting current. The graphs labeled as voltammograms are shown in Figure 5. As can be seen, the received voltammograms have a flattened segment in their curves. This section represents the value of the limiting current. In the considered cases, the flat-test section was not reached. The most representative limiting current is for the lowest velocity of the electrolyte. In this case, the Reynolds number is about 900 and the flow is laminar. In the case of higher fluid velocities, represented by Reynolds numbers between 1800 and 3000, a turbulent flow exists. This is because the critical Reynolds number for flow through the mini-channels is much lower than that of conventional channels [25]. Flow fluctuations that lead to varying turbulence intensities may be the cause of the diminished flat segment in voltammograms. Fluctuations in electrolyte flow through the long mini-channels were also observed by the authors of [21,24]. In both works, the authors reported an increase in the mass transfer coefficient in the entrance section of the channel, then a decrease, followed by a slow increase and a final decrease in the exit part of the mini-channel. Fluctuations in limiting current [24] and, hence the mass transfer coefficient, may indicate turbulence along the length of the channel.

Another cause of the indistinct plateau segment representing the limiting current may be the kind of electrode material used. The latest research [26] shows that electrodes with different metal composition are not suitable at high electrolyte velocities. The best material for electrode is gold or platinum. Use of electrodes with higher nickel content also results in well-defined plateau sections. In the present work, the content of nickel in the electrode material was high, but not high enough to ensure distinct plateau region under turbulent flow conditions.

**Figure 6:**
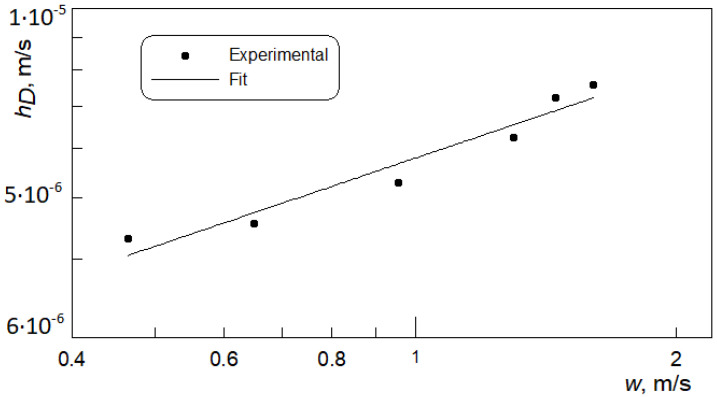
Experimental measured mass transfer coefficients vs. mean electrolyte velocity.

Measurements were performed in the electrolyte velocity range of 0.46 to 1.61 m/s. Six test points were considered. On the basis of the resulting-limiting currents and the measurements of the ion concentration in the working electrolyte, the values of mass transfer coefficients were calculated according to Equation (3). Figure 6 includes the dependence of mass transfer coefficient *h_D_* on mean electrolyte velocity *w*. An increase in *h_D_* with the fluid velocity is observed. The reason for this is attributed to increased turbulences in the fluid flow that results in the enhancement of mass transfer processes.

The measurement uncertainty in arriving at the mass transfer coefficient was determined according to the general rule of summing in quadrature the uncertainties of the following measured parameters: limiting current, cathode surface, and ion concentration (Equation (3)). The equation for mass transfer coefficient uncertainty has a form:

(7) $$\frac{\delta h_{D}}{h_{D}}={\left({\left(\frac{\delta I_{p}}{I_{p}}\right)}^{2}+{\left(\frac{\delta A}{A}\right)}^{2}+{\left(\frac{\delta C_{b}}{C_{b}}\right)}^{2}\right)}^{1/2}$$

where δ*I_p_*, δ*A*, δ*C_b_*—are the mean uncertainties of the following measured parameters: limiting current, cathode surface and ion concentration. From this equation, the uncertainty of *h_D_* determination was evaluated as 2.5%.

The results of investigations of mass transfer coefficients may also be presented in a nondimensional form, the Chilton–Colburn coefficient for mass transfer *j_M_* [27], which is defined as:

(8) $$j_{M}={\mathrm{St}}_{M}{\mathrm{Sc}}^{2/3},$$

where St_M_—Stanton number for mass transfer, Sc—Schmidt number; ${\mathrm{St}}_{M}=h_{D}/w$; Sc = *ν/D*; *ν, D—*kinematic viscosity and the diffusion coefficient of the electrolyte, respectively. In the present experiment, the Sc number was 1590. 

Based on the regression analysis, the received measurement results were correlated to the form:

(9) jM=0.068Re−0.589,

with the coefficient of determination *r* = 0.9406. The Reynolds number is equal to Re=wdH/ν.

**Figure 7:**
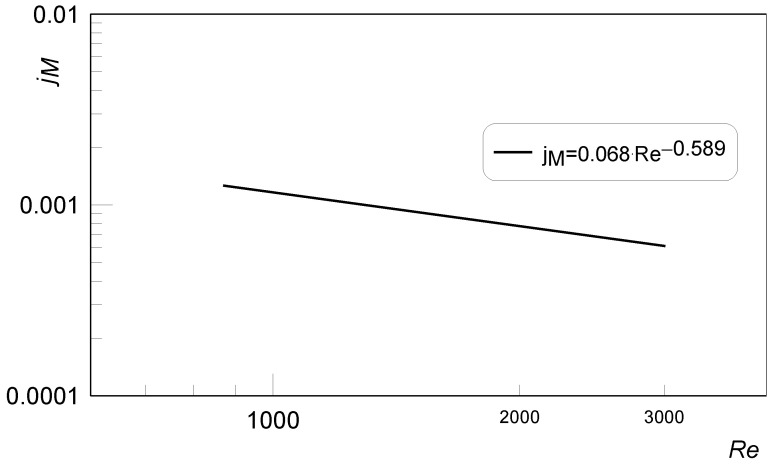
Chilton–Colburn coefficient vs. Reynolds number for mas transfer during the fluid flow through a system of long square mini-channels.

Re numbers were in a range from 870 to 3010. Material properties of the electrolyte, kinematic viscosity and diffusion coefficient, were determined for a measurement’s temperature of a 22 °C. The results of the *j_M_* coefficients determination are presented in Figure 7. 

The mass transfer results received during the experiment based on the limiting current method allow for discussion of the heat transfer phenomena in a system of square long mini-channels. Using Chilton–Colburn analogy of mass/heat transfer in the form:

*j_M_ = j_H_*, (10)

where *j_H_*—Chilton-Colburn coefficient for heat transfer, the heat transfer coefficients characterizing the convective fluid flow through a system of long, square mini-channels may be determined. Considering that:

(11) $$j_{H}={\mathrm{St}}_{H}{\mathrm{Pr}}^{2/3},$$

where St_H_—Stanton number for heat transfer, Pr—Prandtl number; ${\mathrm{St}}_{H}=h/\left(w\rho c_{p}\right)$; $Pr=\nu \rho c_{p}/k$*; h—*heat transfer coefficient; *ρ, c_p_*, *k—*density, specific heat and thermal conductivity of the fluid, respectively, the equation of dimensionless heat transfer coefficient, Nusselst number Nu, has a form:

(12) $$\mathrm{Nu}=0.068Re^{0.411}{\mathrm{Pr}}^{1/3},$$

where $\mathrm{Nu}=hd_{H}/k$.

The results of Nusselt numbers calculations according to Equation (12) were compared with the Gnielinski correlation [28] (Equation (13)) valid for the range of Reynolds numbers up to 2300. It is given by:

(13) $$\mathrm{Nu}={\left\{3.66^{3}+0.7^{3}+{\left[1.615\sqrt[{3}]{\mathrm{RePr}\frac{d}{L}}-0.7\right]}^{3}+{\left[{\left(\frac{2}{1+22Pr}\right)}^{1/6}{\left(RePr\frac{d}{L}\right)}^{1/2}\right]}^{3}\right\}}^{1/3},$$

**Figure 8:**
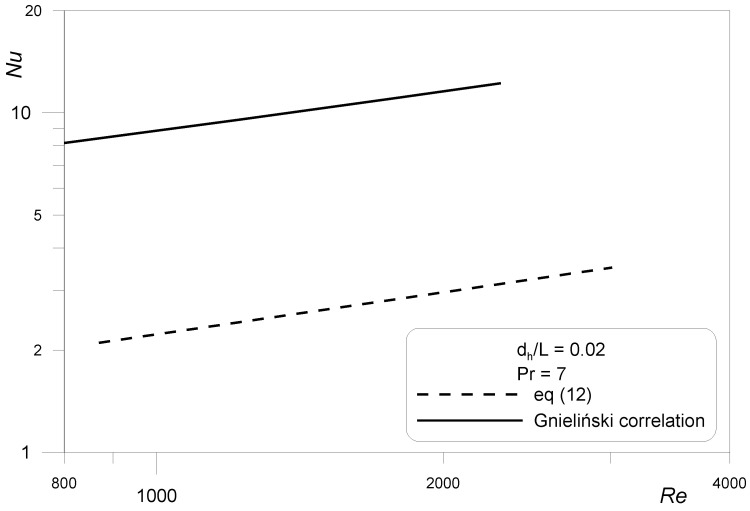
Nusselt numbers from Chilton–Colburn analogy in a square mini-channels system compared with the Gnielinski correlation valid for conventional tubes.

This correlation has been received on the basis of a large database of results of experimental thermal investigations of heat transfer in conventional tubes. The comparison is shown in Figure 8. The graph of Gnielinski correlation represents a case of constant wall temperature boundary condition that corresponds to the situation of constant ion concentration at the mass transfer surface in the limiting current method. 

As can be seen, the mass/heat transfer analogy results for square mini-channels are lower than those for the Gnielinski correlation consistent with the conventional theory. When analyzing the literature data, it can be noticed that researchers, in their thermal experiments, found both higher or lower Nusselt numbers for convective fluid flow through micro- and mini-channels in comparison to conventional cases. The lower results were reported in [29,30,31,32].

The results received in the present study of heat transfer coefficients characterizing the convective fluid flow through a system of long, square mini-channels could be useful in the design and analysis of a mini heat exchangers operation.

## 4. Conclusions

In the present study, the results of the investigations of mass/heat transfer processes occurring in the system of long square mini-channels have been presented. The limiting current technique and the mass/heat transfer analogy method have been applied for determination mass and heat transfer coefficients. On the basis of the received results some concluding remarks can be formulated:

- The material of the cathode modeling the heat transfer surface has an impact on the flat part of the voltammogram and limiting current value determination may be difficult, especially at higher electrolyte velocities.
- As the considered mini-channels are long, the change of the ion concentration along the channel length should be considered in the mass transfer coefficient calculations.
- The received heat transfer coefficients are lower than conventional channels. The specific characteristics of the channel cross section, square and small dimensions, may be the cause of this condition.

## Data Availability

Not applicable.

## Nomenclature

*A*	cathode surface area, m^2^
*c_p_*	specific heat, J/(kgK)
*C*	ion concentration, kmol/m^3^
*d_H_*	hydraulic diameter, m
*D*	diffusion coefficient, m^2^
*F*	Faraday constant, As/kmol
*h*	heat transfer coefficient, W/(m^2^K)
*h_M_*	mass transfeer coefficient, m/s
*I*	current intensity, A
*I_p_*	limiting current, A
*j_H_, j_M_*	Chiltion–Colburn fcoefficient for heat and mass transfer, respectively
*k*	thermal conductivity, W/(mK)
*L*	channel length, m
*n*	valence charge of reacting ions
*N*	molar flux density, kmol/(m^2^s)
Nu	Nusselt number
Pr	Prandtl number
Re	Reynolds number
Sc	Schmidt number
Sh	Sherwood number
St_H_	Stanton number for heat transfer
St_M_	Stanton number for mass transfer
*U*	voltage, V
$\dot{V}$	volumetric flow rate, m^3^/s
*w*	mean flow velocity, m/s
*ν*	kinematic viscosity, m^2^/s
*ρ*	deensity, kg/m^3^
